# Revisiting the Role of γδ T Cells in Anti-CMV Immune Response after Transplantation

**DOI:** 10.3390/v13061031

**Published:** 2021-05-29

**Authors:** Ahmed Gaballa, Faisal Alagrafi, Michael Uhlin, Arwen Stikvoort

**Affiliations:** 1 Department of Clinical Science, Intervention and Technology, Karolinska Institutet, 141 52 Stockholm, Sweden; faisal.alagrafi@ki.se (F.A.); michael.uhlin@ki.se (M.U.); arwen.stikvoort@ki.se (A.S.); 2 Department of Biochemistry and Molecular Biology, National Liver Institute, Menoufia University, Shebin Elkom 51132, Egypt; 3 National Center for Biotechnology, King Abdulaziz City for Science and Technology (KACST), Riyadh 11442, Saudi Arabia; 4 Department of Applied Physics, Science for Life Laboratory, Royal Institute of Technology, 141 52 Stockholm, Sweden; 5 Department of Immunology and Transfusion Medicine, Karolinska University Hospital, 141 52 Stockholm, Sweden

**Keywords:** CMV, gamma delta, transplantation

## Abstract

Gamma delta (γδ) T cells form an unconventional subset of T lymphocytes that express a T cell receptor (TCR) consisting of γ and δ chains. Unlike conventional αβ T cells, γδ T cells share the immune signature of both the innate and the adaptive immunity. These features allow γδ T cells to act in front-line defense against infections and tumors, rendering them an attractive target for immunotherapy. The role of γδ T cells in the immune response to cytomegalovirus (CMV) has been the focus of intense research for several years, particularly in the context of transplantation, as CMV reactivation remains a major cause of transplant-related morbidity and mortality. Therefore, a better understanding of the mechanisms that underlie CMV immune responses could enable the design of novel γδ T cell-based therapeutic approaches. In this regard, the advent of next-generation sequencing (NGS) and single-cell TCR sequencing have allowed in-depth characterization of CMV-induced TCR repertoire changes. In this review, we try to shed light on recent findings addressing the adaptive role of γδ T cells in CMV immunosurveillance and revisit CMV-induced TCR reshaping in the era of NGS. Finally, we will demonstrate the favorable and unfavorable effects of CMV reactive γδ T cells post-transplantation.

## 1. Introduction

Human cytomegalovirus (CMV), also known as human herpesvirus 5, is a 235 kb double-stranded DNA virus that belongs to the β-herpes virus family. CMV is a widespread virus with a seroprevalence that ranges from 45–100% in the population [1]. Evolutionarily, CMV has adapted to its human host by developing multiple immunomodulatory mechanisms to evade immune recognition. As a result of the robust immune response in immunocompetent individuals, CMV results in an asymptomatic lifelong latent infection [2], whereas in immunocompromised patients, CMV reactivation is often associated with severe morbidity and mortality [3]. The impact of CMV is particularly important in the context of transplantation, as CMV infection/reactivation represents a common infectious complication. Multiple studies indicated CMV seropositivity of recipient and/or donor to be associated with adverse prognosis in terms of reduced overall survival post-transplantation [4,5,6]. Furthermore, CMV infection has been associated with several unfavorable outcomes such as graft rejection [7] and posttransplant lymphoproliferative disorders [8]. The immune response to CMV involves both the innate and the adaptive immune systems. Although most of the credit goes to αβ T, B, and NK cells, accumulating evidence supports a potential protective role of γδ T cells.

Gamma delta (γδ) T cells are unconventional T lymphocytes that express a T cell receptor (TCR) formed of a γ and a δ chain [9]. Alongside B and αβ T cells, γδ T cells have coevolved in vertebrates for several million years and have been implicated in antimicrobial and antitumor immune surveillance [10]. In contrast to conventional αβ T cells, the majority of γδ T cells do not express CD4 or CD8 molecules. Furthermore, antigen recognition by the TCR does not require major histocompatibility complex (MHC) molecules. Additionally, γδ T cells can recognize a wide range of structurally different ligands such as membrane protein, phospholipids, soluble protein, and small peptides and can be activated in both TCR-dependent and independent manners, acting as a bridge between innate and adaptive immunity [11].

Although human γδ T cells account for only 1–10% of circulating T cells in adults, they constitute larger proportions of resident T cells in other tissue compartments such as skin, intestinal mucosa, and the female reproductive system [12]. Moreover, γδ T cells are heterogeneous with different functional capabilities. As a principal function, γδ T cells are key players in lymphoid stress surveillance, providing a rapid antiviral, antitumor immune response. 

## 2. Receptors and Activating Ligands Potentially Involved in CMV Immune Response

Human γδ T cells are classified according to their δ chain expression into two major subsets; Vδ2+ subset and Vδ2^neg^ subset. The latter subset predominantly consists of Vδ1+ cells [13]. Here, we will focus on the receptors expressed by the Vδ1+ (Vδ2^neg^) T cells that are primarily thought to be involved in CMV recognition. 

Vδ2^neg^ γδ T cells are dominated by Vδ1+ T cells, which account for approximately 50% of γδ T cells in fetal blood but represent only a minor proportion of the circulating total γδ T cells in adults. Vδ1+ T cells are involved in lymphoid stress surveillance as they can recognize transformed or infected cells in TCR-dependent or independent mechanisms. Although several efforts have been conducted (Table 1), the exact receptors and ligands implicated in CMV recognition are still enigmatic.

**Figure 1:**
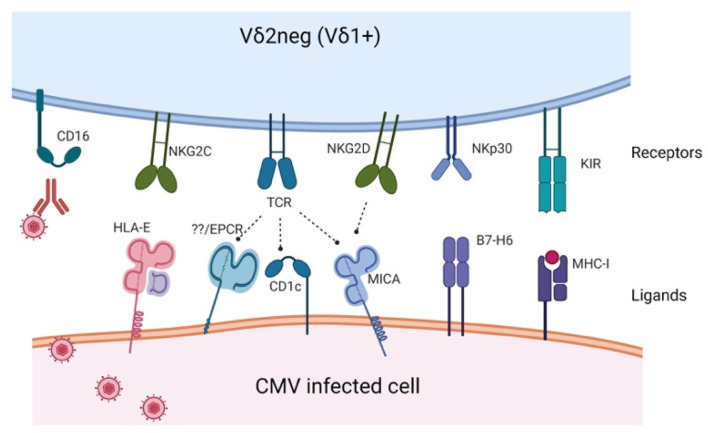
Receptors and ligands that are potentially involved in human cytomegalovirus (CMV) immune response by γδ T cells (created with BioRender.com, April 2021).

In contrast to the semi-invariant Vγ9Vδ2 TCR, Vδ1 TCR recognizes a wider range of antigens (Figure 1), including lipid antigens presented by MHC-like molecules, CD1c and CD1d [25]. Additionally, Vδ1+ TCR can recognize stress-inducible molecules such as MHC class I-related chain A (MICA), though in a much lower affinity (1000-fold lower) compared to natural killer group 2D (NKG2D) [17]. Recently, Wilcox and coworkers have identified endothelial protein C receptor (EPCR) as a possible ligand that mediates CMV recognition by Vγ4Vδ5 T cells [19]. Notably, the activating ligands implicated in CMV immune response and the mechanism by which they recognize CMV are very complex and still elusive [26]. Identifying such ligands would therefore be crucial to unleashing the full immunotherapeutic capabilities of γδ T cells and would enable in vitro expansion of CMV specific cells on a large scale sufficient for the use in adoptive immunotherapy. 

In addition to TCR-mediated recognition, Vδ1 γδ T cells can rapidly recognize transformed or infected cells through TCR-independent mechanisms. Besides pattern recognition receptors [27], Vδ1 T cells are equipped with other receptors such as NKG2D, a C-type, lectin-like dimeric activating receptor [9] which can recognize several stress-induced self-ligands such as MHC class I-related chain A/B (MICA/B) and UL16 binding protein (ULBP) that are upregulated in stressed and infected cells [21]. Nevertheless, whether this receptor is implicated in CMV immune response is not clear as CMV can evade recognition by NKG2D by inhibiting the expression of their ligands [2]. 

In addition to NKG2D, γδ T cells express NKG2C, a receptor that binds the non-classical HLA-E molecule. Similar to NK cells, it has been postulated that NKG2C+ γδ T cells can recognize CMV-derived UL40 peptides in the context of the HLA-E molecule [22]. Studies have shown increased expression of CD16 in γδ T cells during CMV infection. CD16 (FcγIII), is a low-affinity receptor that binds the constant region of immunoglobulin. Thus, they can mediate the recognition and killing of opsonized target cells through antibody-dependent cellular cytotoxicity (ADCC) without the need for TCR engagement [28]. CMV-induced downregulation of HLA class I as an immune evasion mechanism can also enhance the cytotoxicity of Vδ1+ cells through activation of killer inhibitory receptors (KIR) [24].

## 3. CMV-Induced Changes in γδ T Cells

### 3.1. Composition Changes

**Figure 2:**
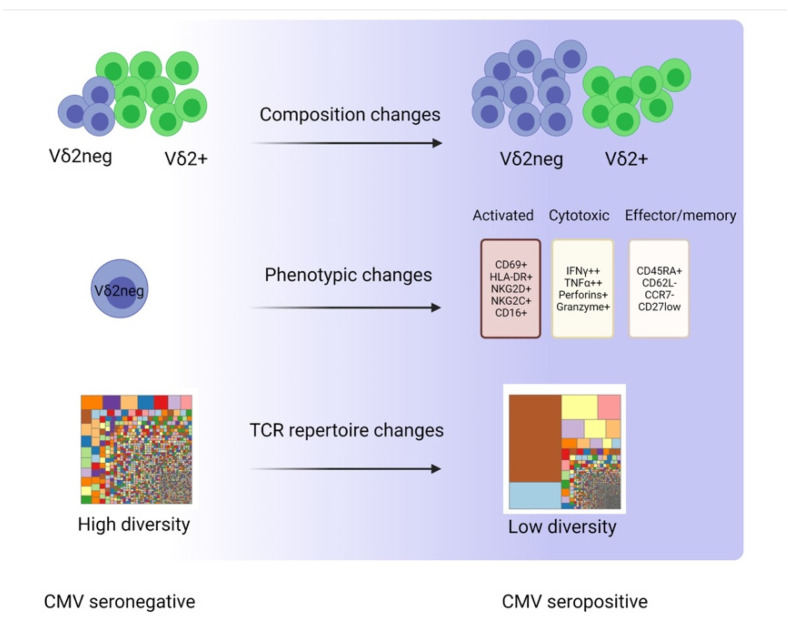
CMV-induced changes in γδ T cells: a representative figure showing the phenotypic, composition, and clonotypic changes in γδ T cells in response to CMV (created with BioRender.com).

Though Vδ2+ γδ T cells represent the predominant γδ T cells in peripheral blood (PB), the massive expansion induced by CMV is characteristically confined to the Vδ2neg subset. Similar to conventional CD8+ T cells, Vδ2^neg^ subsets, particularly the Vδ1+ subset, undergo a remarkable expansion that leads to perturbation of the Vδ2+/Vδ2^neg^ ratio in the PB (Figure 2). Interestingly, in some CMV seropositive patients, the proportion of Vδ1+ cells can reach up to 50% of circulating T cells [29]. In addition to the CMV-induced Vδ1+ expansion, additional Vδ2neg populations including Vδ3+ and Vδ5+ subsets are also involved, suggesting a common feature between those subsets. In fact, the CMV-induced alteration in the γδ T cell composition in the PB was reported as early as 1999 in kidney transplant patients [30,31]. Since then, the accumulating bulk of evidence from different transplant settings has not only corroborated these results but also indicated that this prominent expansion is also stable over years (Table 2), suggesting a specific blood signature of CMV infection [32].

### 3.2. Phenotypic Changes

In addition to the CMV-induced expansion, γδ T cells express several markers consistent with an activation phenotype. Several studies have indicated increased expression of CD69 and HLA-DR but not CD25 [29]. Furthermore, CMV responsive γδ T cells displayed increased expression of markers associated with enhanced cytotoxic capabilities such as perforin and granzyme B [29,39]. Similar to CD8+ T cells, CMV-induced activation and proliferation in γδ T cells were also accompanied by differentiation suggestive of memory development. Numerous studies in both healthy subjects and renal transplant patients showed that a large proportion of CMV-reactive γδ T cells display markers associated with the terminally differentiated phenotype (CD45RA+, CCR7−, CD62L−) [29]. In more recent studies, differential expression of CD27 was used to define the memory phenotype of Vδ1 T cells. The proportion of naïve-like Vδ1 cells, defined as Vδ1+CD27+, was higher in CMV seronegative healthy individuals, whereas a higher proportion of Vδ1+ cells that are CD27^low^ alongside clonal focusing was observed in CMV seropositive individuals, suggesting an effector/memory phenotype [45,46].

### 3.3. TCR Repertoire Changes

The differentiation and maturation of T cell progenitors into either αβ or γδ T cell lineages is a complex process that takes place within the thymus [25]. Like αβ T cells, γδ T cells undergo somatic rearrangement of their variable (V), diversity (D), and joining (J) gene segments of the *TRD* locus (located on chromosome 14 within the *TRA* gene loci) and the V and J gene segments for the *TRG* locus found on chromosome 7 [47]. Though this process can theoretically result in high γδ TCR repertoire diversity, the predominant subset of adult circulating γδ T cells (Vγ9Vδ2) displays a semi-invariant TCR with only limited diversity, suggesting a role of phosphoantigen in reshaping γδ T cells’ repertoire throughout life [48].

The advent of next-generation sequencing (NGS) alongside the development of analysis packages that do not require prior extensive knowledge of bioinformatics has allowed researchers to answer complex questions that would have been otherwise impossible. Recent evidence indicated that the TCR δ chain repertoire is more diverse yet comprised of private (less public) sequences, unlike the TCR γ chain repertoire that is more public and relatively less diverse [49]. Likewise, differences in TCR repertoire have also been reported between γδ T cell subsets. While Vδ2+ cells display a TCR repertoire of low/intermediate diversity throughout life, the TCR repertoire of Vδ2^neg^ cells appears more diverse initially (at birth) but becomes more clonally focused later during adulthood [46]. This remarkable reshaping of the Vδ2^neg^ TCR repertoire has been attributed to CMV infection. Several studies have confirmed the restricted γδ TCR repertoire in CMV seropositive healthy donors and transplant patients using different techniques. For instance, Pitard et al. analyzed TCR-δ chain CDR3-length distribution by Immunoscope, while Knight and colleagues showed similar findings in CMV seropositive healthy subjects and HSCT patients using the TCR spectratyping method [32,44]. 

Several recent studies have used NGS and single-cell TCR sequencing to explore in-depth the TCR repertoire perturbation in response to CMV infection in healthy subjects and post-HSCT. Their results revealed significant clonal expansion, suggesting that CMV-reactive γδ T cells respond to a driving antigen (CMV) by the clonotypic expansion of a few clones (Figure 2). Furthermore, CMV characteristically altered Vγ gene segment usage in the form of a marked reduction in Vγ9 and increased utilization of Vγ2, Vγ4, and Vγ5 gene segments [46,49]. Using NGS, our group has investigated the impact of CMV seropositivity on the *TRG* repertoire of 20 PB stem cell donor grafts. Our findings corroborated previous studies showing CMV-associated clonal perturbations and bias in V-J gene segment usage [50]. Altogether, the reshaping of γδ TCR repertoire by CMV can be regarded as a specific blood signature of CMV infection. Notably, this CMV-driven clonal proliferation of individual virus-reactive γδ TCR sequences has provided strong evidence for adaptive anti-viral γδ T cell immune responses, as will be discussed in the next section.

## 4. Adaptive-Like Immune Response to CMV Infection

The hallmark of the adaptive immune cells centers on their ability to develop immunologic memory alongside clonal selection and proliferation upon recognition of cognate antigen [45,51]. Unlike conventional αβ T cells, the ability of γδ T cells to mount an adaptive immune response has remained a matter of debate. γδ T cells are classically described as a “bridge” between innate and adaptive cells with more innate features. Analogous to mucosal-associated invariant T (MAIT) cells and invariant natural killer T (iNKT) cells, the predominant fraction of PB γδ T cells express a TCR (Vγ9+ Vδ2+) that displays semi-invariant features with limited diversity and antigen specificity. Furthermore, the ability of γδ T cells to respond promptly to stress-induced signals in a TCR-independent way fits well with the innate-like paradigm [45].

Unlike their Vδ2+ counterparts, the immune response of Vδ2^neg^ γδ T cells to CMV has provided an attractive model to study the adaptive-like features of these cells. By comparing phenotypic and clonotypic changes in γδ T cells between CMV seropositive and seronegative healthy subjects, Pitard et al. demonstrated that differentiation of Vδ2^neg^ cells from naïve to effector/memory phenotype was associated with marked repertoire restriction, strongly indicating CMV-driven clonal selection. Furthermore, upon secondary challenge with CMV, Vδ2^neg^ cells from seropositive donors mounted a quicker anti-CMV immune response. Together, these findings are consistent with an adaptive immune response [32]. In a recent study, Davey and others showed that CMV-induced Vδ1 differentiation from naïve-like (CD27 high) to effector/memory (CD27 low) phenotype was associated with increased clonality. Furthermore, memory-like Vδ1 cells displayed enhanced response to TCR and cytokine stimuli, suggesting adaptive rather than innate immune response [46].

The adaptive immune response to CMV has not been limited to the Vδ1+ subset but has been reported in other distinct γδ T cell subsets. In another study by Davey et al., a distinct subset of Vδ2+ cells was identified. Nevertheless, unlike Vγ9+ Vδ2+ cells, this novel subset preferentially pairs to non Vγ9 (Vγ9-Vδ2+) and was responsive to TCR but not phosphoantigen stimulation. Importantly, they showed that Vγ9-Vδ2+ cells displayed clonal expansion in response to CMV infection in kidney transplant patients, suggesting adaptive-like biology [51]. In bone marrow stem cell grafts, we showed increased proportions of CD8+ γδ T cells in grafts from CMV-seropositive donors. This CMV-induced expansion was associated with differentiation towards effector/memory phenotype. TRG immune sequencing and spectratype analysis of the TCR repertoire indicated more restricted repertoire in CD8+ γδ T cells compared to CD8-γδ T cells from the same donor. Additionally, CD8+ γδ T cells differentially respond to TCR stimulation and cytokines [52]. Overall, these findings provided a piece of strong evidence for the adaptive immune response of CD8+ γδ T cells.

Although the adaptive-like clonotypic response of γδ T cells to CMV has been widely shown, conflicting findings were reported in a small cohort of lung transplant recipients after CMV reactivation. While CMV induced the expansion of the Vδ1+ subset, which also displayed phenotypic changes suggestive of typical memory differentiation, the analysis of TCR revealed a surprisingly diverse repertoire. Interestingly, Vδ1+ expansion was also associated with increased expression of the NKG2C receptor [22]. Although the adaptive role of NKG2C in CMV immune response has been previously described in NK cells, very little is known about its role in γδ T cells. NKG2C has been shown to recognize the CMV-derived UL40 peptide presented by HLA-E molecule (a monomorphic non-classical MHC class I molecule) [53]. These results suggest that γδ T cells can mount an unconventional adaptive immune response in a TCR-independent manner.

## 5. Effects of CMV Reactive γδ T Cells after Transplantation

### 5.1. Favorable Effects

#### 5.1.1. Anti-CMV Protective Role

Several studies have demonstrated a remarkable expansion of Vδ2^neg^ γδ T cells during CMV infection in healthy subjects, solid organ transplants, as well as post-HSCT. Moreover, this marked expansion was specific for CMV infection and not other viral infections (such as HSV, VZV, EBV, and influenza) and was concomitant with the resolution of CMV [34,35,36]. Experimentally, Vδ2^neg^ cells isolated from CMV-seropositive donors were able to specifically lyse CMV-infected targets [44]. Altogether, these findings strongly support the protective immune responses to CMV reactivation and CMV resolution.

Notably, monitoring γδ T cell kinetics in the PB during the course of the infection allows the prediction of CMV infection resolution, which would help to adjust the duration and the dose of antiviral drugs. In a three-year prospective study of the kinetics of γδ T cells in 190 patients undergoing kidney allograft transplantation, Lafarge et al. showed delayed γδ T expansion to be associated with persistent CMV viremia and severe symptoms [3]. Likewise, Kaminski and coworkers showed that expansion of 0.06% Vδ2neg γδ T cells per day after infection correlated with resolution of CMV viremia in kidney transplants [36]. In the context of allogeneic HSCT, the protective antiviral role of Vδ2neg γδ T cells has been reported. In a cohort of 40 HSCT patients, Knight et al. showed significant increase in the numbers of Vδ2neg γδ T cells after CMV reactivation which persisted for long durations, suggesting a role in CMV immune response [44]. In line with this, we demonstrated in a recent meta-analysis of five different studies that higher numbers of γδ T cells post-HSCT were associated with less frequent viral infections [54]. Altogether, these data support that γδ T cells play a protective antiviral role in the HSCT setting comparable to that reported in the solid organ transplantation setting. 

Though Vγ9Vδ2 γδ T cells are largely known to not be involved in the immune response to CMV, it has been postulated that their antiviral activity could be enhanced by CMV. Daguzan and colleagues investigated the impact of zoledronate on cytokine response by Vγ9Vδ2 γδ T cells in the presence or absence of CMV infection. Their results showed that fibroblasts treated with ZOL only activated IFN-γ but not TNF secretion by Vγ9Vδ2 γδ T cells, whereas in CMV-infected fibroblasts the same treatment led to TNF secretion also. These findings indicated a synergistic effect of CMV, mostly through increasing the level of phosphoantigens by different mechanisms [55].

#### 5.1.2. Anti-Tumor Role

Although the link between CMV reactivation and relapse after HSCT has been reported since 1986 [56], it is still a matter of debate whether CMV reactivation has a beneficial antileukemic effect, as several contradicting results have been published. For instance, in a large registry study that involved 9469 patients from the CIBMTR database, Teira et al. found no correlation between CMV reactivation and relapse incidence [57]. On the contrary, Elmaagacli and Koldehoff showed in a recent meta-analysis that included 8511 AML patients from 6 different studies that CMV reactivation was associated with a reduced risk of relapse in 5 out of the 6 studies [58].

Notably, the antileukemic effect of CMV reactivation has been reported mainly in the context of AML [59,60,61,62] and to some extent CML [63], while no or very few studies have reported similar effects in other types of hematological malignancies. Furthermore, the results varied regarding the type of conditioning. While Manjappa et al. showed reduced AML relapse incidence after CMV reactivation in patients transplanted with myeloablative conditioning [64], another group showed similar effects but only in patients that received reduced-intensity conditioning [65].

Importantly, while CMV reactivation can reduce the risk of relapse in AML patients, its impact on the overall outcome is still unsettled. In this regard, multiple large studies have reported increased non-relapse mortality (NRM) rate and decreased overall survival (OS) after CMV reactivation [6,57], whereas others found no significant association [66] or even improved OS [59,62]. 

Of note is that pooling results from different studies should be carefully interpreted given the variations in the methods used to detect CMV viremia and hence the cutoff values, besides the differences in CMV treatment protocols. Furthermore, the lack of consensus regarding the definition of CMV reactivation and the reporting of CMV reactivation as a binary variable (Yes/No) might lead to differences among studies. To overcome this limitation, Leserer et al. assessed the clinical outcomes in 705 HSCT patients using CMV kinetic models. By stratifying patients into low, intermediate, or high CMV peak titer cohorts, they showed that patients in the high-CMV peak titer group had increased NRM and reduced OS concomitant with delayed early immune reconstitution. Interestingly, the intermediate-CMV peak titer group was associated with reduced relapse alongside adequate immune reconstitution pattern as indicated by increased T cell numbers [67]. 

Importantly, it is not clear whether CMV per se can directly kill leukemic blasts since CMV can act in favor of tumors by preventing apoptosis of infected cells [68]. Nevertheless, Koldehoff and coworkers showed experimentally that human CMV can induce apoptosis of acute leukemia cell lines via a caspase-dependent pathway, arguing for a virus-versus-tumor effect [69]. Regardless of this debate, it is more evident that the favorable impact following CMV reactivation is mediated by the activation of innate and adaptive immune cells that share reactivity to CMV and tumor cells. T cells appear to play a key role in this complex relation, as several retrospective studies have failed to show any significant association between relapse and CMV reactivation in HSCT patients that received T cell-depleting therapy [70,71]. Likewise, the anti-leukemic effect of NK cells has been postulated to be enhanced during CMV reactivation. The underlying mechanism includes CMV-driven expansion of NKG2C+ NK cells, leading to a shift from expressing the inhibitory NKG2A receptor to the activating NKG2C receptor, which recognizes leukemic blasts through binding HLA-E molecules [72,73]. 

Similarly, the protective effect of CMV reactive γδ T cells was not limited to their ability to lyse CMV-infected cells but extended to demonstrate their potential contribution in the anti-tumor immune response. Nevertheless, the evidence for their ability to exert a protective anti-tumor role is still a matter of debate. In support of the postulated antitumor function, Halary et al. demonstrated that γδ T cells can react to both CMV infected and HT29 colon cancer cells in a TCR-dependent manner, suggesting cross-reactivity to self-antigens that are shared between CMV-infected and tumor cells [74]. In line with these findings, a recent study showed that endothelial protein receptor C (EPCR) is recognized by Vγ4Vδ5 T cells. Interestingly, EPCR was found to be expressed by both epithelial tumors and CMV-infected endothelial cells, supporting the hypothesis of cross-reactivity [19]. In a longitudinal case-control study of kidney transplant patients, recipients with increased Vδ2^neg^ γδ T cells following CMV reactivation had a lower incidence of cancers [40].

In the HSCT setting, Scheper et al. showed that Vδ2^neg^ γδ T cells isolated from CMV-reactivating patients could specifically recognize both CMV-infected cells and hematological tumors such as lymphoma (Daudi), leukemia, myeloma cell lines, and primary AML blasts [43]. In line with this, a study that included 153 partially mismatched related HSCT patients showed improved leukemia-free survival and overall survival in patients with increased Vδ1+ γδ T cell numbers in PB [75]. Conversely, Knight et al. showed that in vitro expanded Vδ1 γδ T cells can recognize and kill glioblastoma independent of CMV [76]. Similarly, γδ cells isolated from a 48-year-old patient who received HSCT for B-CLL showed reactivity against CMV target cells, but not against the relapsing lymphoma cells, arguing against an antitumor role of CMV-reactive γδ T cells [42]. Furthermore, inhibition of NKG2D ligands by CMV can negatively affect the antitumor response. Those contradicting results reflect the complexity of the landscape and could be due to the heterogeneity between different studies and within the same study concerning the donor type, underlying disease, and its stage, etc. Therefore, meta-analysis and multicenter studies should be conducted to provide an estimate of the overall effect.

### 5.2. Unfavorable Effects

In the context of HSCT, acute GVHD (aGVHD) and CMV reactivation are both closely associated, and evidence for a bidirectional relation has been suggested [77]. It has been shown that CMV reactivation is implicated in the initiation of aGVHD. On the other hand, aGVHD or its treatment increases the risk of CMV reactivation [78,79]. Though the underpinning mechanisms are still poorly understood, a complex interplay between different immune cells is suggested. In a recent study, we showed an increased cumulative incidence of aGVHD II-III in HSCT patients that received stem cell grafts containing higher proportions of CD8+ γδ T cells. Mixed lymphocyte reactions indicated potential alloreactive response by CD8+ γδ T cells, suggesting a potential role in the immunopathology of aGVHD [80]. In a subsequent study, we showed that the proportions of CD8+ γδ T cells were significantly higher in bone marrow stem cell grafts from CMV-seropositive donors, suggesting a potential role in CMV immune response [52]. Combined, these data provide the basis for understanding the relationship between CMV and GVHD after HSCT. Though the mechanisms are still undefined, we propose that CMV and aGVHD could be mediated by cross-reactivity of CMV-specific CD8+ γδ T cells to alloantigens. Further investigations are needed to alleviate the immunobiology of CD8+ γδ T cells and the role of the CD8 molecule in this context.

In solid organ transplantation, graft rejection represents a major complication. Numerous studies have investigated the impact of CMV on graft rejection, suggesting an unfavorable role possibly mediated by cross-reactivity of virus-specific T cells to alloantigens [81,82]. Nevertheless, more recent studies have shown opposite results suggesting that the impact can differ under certain conditions [83,84]. In this regard, little is known about the role of CMV-reactive γδ T cells and the results from available studies are contradicting. In a study that included 32 adult LTX patients, gene expression profile and immunophenotyping post-transplantation showed an increased level of Vδ1+ γδ T cells in operationally tolerant liver recipients [85]. Another study in pediatric LTX patients showed that the intragraft Vδ1+/Vδ2+ ratio was significantly increased in the tolerant patient group [86]. Given that CMV reactivation is very common after LTX and that Vδ1+ expansion is mainly driven by CMV, it is reasonable to speculate that CMV reactive Vδ1+ cells are an important player in this context. On the other hand, Bachelet et al. showed in KTX recipients that CMV-responsive γδ T cells induce allograft dysfunction in an antibody-dependent cellular cytotoxicity manner. The mechanism possibly involves the recognition of donor-specific antibodies by CD16 receptors which are highly expressed by CMV-responsive γδ T cells [87].

## 6. Concluding Remarks

The past two decades have witnessed a growing interest in γδ T cells. The immunological capabilities of these unconventional cells have been intensively explored. However, more efforts aimed at unraveling the immunobiological features of different γδ T cell subsets are warranted to effectively exploit their full immunotherapeutic potential. 

During transplantation, CMV reactivation has been associated with unfavorable outcomes and therefore, preemptive antiviral therapy is crucial. However, excessive use of antiviral drugs is associated with severe toxicities and an increased risk of developing drug resistance. As an alternative to antiviral treatment, immunotherapeutic approaches based on the use of CMV-specific cytotoxic T lymphocytes (CTLs) have been investigated. Nevertheless, the benefit of such approaches is limited by the HLA compatibility, which restricts the possibility to use universal donors for CTL production. Luckily, the non-classical mode of action of γδ T cells being mainly HLA-unrestricted permits their use across HLA barriers. Nevertheless, achieving this goal is still hampered by the poor understanding of the ligands involved. A better understanding of this complex network is still needed to be able to foster the design of new γδ T cell-based therapies.

## Figures and Tables

**Table 1:** A list of receptors and corresponding activating ligands for γδ T cells.

Receptor	Ligand	Study
Vδ1 TCR	CD1c, CD1d, MICA, PE,	Zeng et al. [14] Roy et al. [15] Uldrich et al. [16] Xu et al. [17]
Vγ4Vδ1 TCR	BTNL3-BTNL8	Melandri et al. [18]
Vγ4Vδ5 TCR	EPCR	Willcox et al. [19]
Vγ8Vδ3 TCR	Annexin A2	Marlin et al. [20]
NKG2D	MICA/B, UL16BP	Silva-Santos et al. [21]
NKG2C	HLA-E	Stankovic et al. [22]
NKp30	B7-H6	Brandt et al. [23]
KIR	HLA-I	Dolstra et al. [24]

TCR: T cell receptor; CD: cluster of differentiation; BTNL: butyrophilin-like 3; EPCR: endothelial protein C receptor; NKG2D/C: natural killer group 2 D/C; MICA: MHC class I-related chain A; KIR: killer cell immunoglobulin-like receptor.

**Table 2:** CMV-induced expansion of Vδ^neg^ γδ T cells in different transplant settings.

Transplant Setting	Expanded γδ Subset	Study
Lung TX	NKG2C+ γδ T cells	Stankovic et al. [22]
Liver TX	Vδ1 γδ T cells	Puig-Pey et al. [33], D´Offizi et al. [34]
Kidney TX	Vδ2^neg^, Vγ9-Vδ2+, Vδ1 and Vδ3, CD16+ γδ T cell	Kaminski et al. [35,36], De´chanet et al. [30,31], Lee et al. [37], Couzi et al. [38,39,40,41]
HSCT	Vδ2^neg^, Vδ1 γδ T cell	Prinz et al. [42], Scheper et al. [43], Knight et al. [44]

TX: transplantation; HSCT: hematopoietic stem cell transplantation.
